# From smoking cessation to physical activity: Can ontology-based methods for automated evidence synthesis generalise across behaviour change domains?

**DOI:** 10.12688/wellcomeopenres.21664.2

**Published:** 2025-03-24

**Authors:** Oscar Castro, Emma Norris, Alison J Wright, Emily Hayes, Ella Howes, Candice Moore, Robert West, Susan Michie

**Affiliations:** 1 Centre for Behaviour Change, University College London, London, England, UK; 2 Future Health Technologies, Singapore-ETH Centre, Campus for Research Excellence and Technological Enterprise (CREATE), Singapore, Singapore; 3 Health Behaviour Change Research Group, Department of Health Sciences, Brunel University London, London, England, UK; 4 Institute of Pharmaceutical Science, King's College London, London, England, UK; 5 Clinical Trials Research Unit, University of Leeds, Leeds, England, UK; 6 Department of Behavioural Science and Health, University College London, London, England, UK

**Keywords:** ontology, taxonomy, classification system, evidence synthesis automation, systematic review, exercise, movement behaviours

## Abstract

**Background:**

Developing behaviour change interventions able to tackle major challenges such as non-communicable diseases or climate change requires effective and efficient use of scientific evidence. The Human Behaviour-Change Project (HBCP) aims to improve evidence synthesis in behavioural science by compiling intervention reports and annotating them with an ontology to train information extraction and prediction algorithms. The HBCP used smoking cessation as the first ‘proof of concept’ domain but intends to extend its methodology to other behaviours. The aims of this paper are to (i) assess the extent to which methods developed for annotating smoking cessation intervention reports were generalisable to a corpus of physical activity evidence, and (ii) describe the steps involved in developing this second HBCP corpus.

**Methods:**

The development of the physical activity corpus involved: (i) reviewing the suitability of smoking cessation codes already used in the HBCP, (ii) defining the selection criteria and scope, (iii) identifying and screening records for inclusion, and (iv) annotating intervention reports using a code set of 200+ entities from the Behaviour Change Intervention Ontology.

**Results:**

Stage 1 highlighted the need to modify the smoking cessation behavioural outcome codes for application to physical activity. One hundred physical activity intervention reports were reviewed, and 11 physical activity experts were consulted to inform the adapted code set. Stage 2 involved narrowing down the scope of the corpus to interventions targeting moderate-to-vigorous physical activity. In stage 3, 111 physical activity intervention reports were identified, which were then annotated in stage 4.

**Conclusions:**

Smoking cessation annotation methods developed as part of the HBCP were mostly transferable to the physical activity domain. However, the codes applied to behavioural outcome variables required adaptations. This paper can help anyone interested in building a body of research to develop automated evidence synthesis methods in physical activity or for other behaviours.

## Plain language summary

The Human Behaviour-Change Project (HBCP) wants to make it easier to gather and analyse information about how to change people's behaviour. To achieve this, the project collects reports about behaviour change interventions, code them based on an ontology (that is, a classification scheme to organise and represent information within a specific area), and use that data to train computer programs to automatically extract information from reports and make predictions. The project started with smoking cessation as a proof of concept but plans to expand to other behaviours. This study investigates how well the methods used for coding smoking cessation intervention reports could be applied to a new behaviour, physical activity, and describes how this new set of data on physical activity intervention reports was created. Building the HBCP physical activity data set involved: (1) checking if the ontology codes used for smoking cessation would work for physical activity, (2) deciding what kind of physical activity reports to include, (3) finding the reports, and (4) coding the reports using the ontology. During step 1 researchers found that some changes were needed to the codes used for smoking cessation, so they analysed 100 physical activity intervention reports and got feedback from experts to update the codes. After stages 2-4, they ended up with 111 physical activity reports coded using the ontology of behaviour change interventions. In conclusion, the methods used for coding smoking cessation intervention reports could mostly be applied to reports about physical activity interventions, but changes were needed in relation to the target behaviour (for example, how behaviour is measured or whether researchers want people to start vs stop doing something). The aim of this report is to help others looking to build a data set to improve ways in which information on behaviour change interventions is gathered and analysed.

## Introduction

The solution to many of the health and environmental challenges that humanity faces today lies in changing people’s behaviour. To achieve this, it is crucial to effectively synthesise and build upon evidence from behaviour change intervention studies cumulatively.

Evidence synthesis refers to the process of compiling relevant studies on a specific topic to answer a particular research question ( Langlois *et al.*, 2018). In recent decades, researchers have placed a stronger emphasis on rigor and accountability of evidence synthesis methods – given birth to the now-standard systematic review methodology – and developed new approaches (e.g., meta-analysis) to statistically combine results from multiple studies ( Thomas, 2024). These advancements have undoubtedly benefited behavioural science and other fields, for example, to help ground decisions on scientific evidence ( Signore & Campagna, 2023). However, many argue that current evidence synthesis methods have inherent limitations ( Moore *et al.*, 2022; Siontis & Ioannidis, 2018) and fail to leverage recent advancements in information and computing sciences which could help address research questions more effectively and efficiently ( Michie & Johnston, 2017; Sharp *et al.*, 2023). Behaviour change evidence is currently extracted from study reports through a manual, lengthy, and error-prone process, without a shared conceptual and linguistic framework in the field to facilitate true cumulative knowledge. This leads to research waste ( Roberts & Ker, 2015), as findings cannot be easily integrated with other research, and represents a missed opportunity to advance our understanding of behaviour change for the better.

The Human Behaviour-Change Project (HBCP) sought to address these limitations by developing an *artificial intelligence*-based Knowledge System that automatically extracts and synthesises information from intervention reports, structured by an *ontology* of *behaviour change interventions* ( Michie *et al.*, 2017; Michie *et al.*, 2020a) (see Table 1 for a glossary of *key terms* used in the article). The HBCP methodology involved building a corpus (i.e., compiling full texts of published intervention reports and *annotating* them according to an ontology) to provide a training and evaluation data set for the Knowledge System’s information extraction and prediction algorithms. The Behaviour Change Intervention Ontology (BCIO) was developed as part of the HBCP to serve as a conceptual framework within which knowledge is structured and formally represented ( Michie *et al.*, 2020b; Norris *et al.*, 2019; Wright *et al.*, 2020). The BCIO offers a set of definitions for describing key *entities* of behavioural interventions and the relationship between those entities, covering intervention content ( Corker *et al.*, 2022; Marques *et al.*, 2023), engagement, population, setting ( Norris *et al.*, 2020), target behaviour ( Schenk *et al.*, 2024), mechanisms of action ( Schenk *et al.*, 2023), and delivery, including mode of delivery ( Marques *et al.*, 2020), source of delivery ( Norris *et al.*, 2021), style of delivery ( Wright *et al.*, 2023) and schedule. This ontology-based approach distinguishes the HBCP from other information extraction efforts in behavioural and social sciences, which typically rely on different approaches such as distant supervision (e.g., Wei *et al.*, 2022), classifiers (e.g., Zielinski & Mutschke, 2017), or neural networks (e.g., Shen *et al.*, 2022) and are mostly focused on extracting data from study abstracts ( Legate *et al.*, 2024; Schmidt *et al.*, 2023), likely due to their greater availability and ease of access compared to full texts.

**Table 1.** Glossary of terms used in the article.

Term	Definition	Source
Annotation	Process of coding selected parts of documents or other resources to identify the presence of ontology entities.	Michie *et al.*, 2017
Artificial intelligence	The practice of building computer programs to perform tasks that a human would reasonably regard as requiring intelligence.	Nilsson, 2014
Behaviour change intervention study	An intervention evaluation study of a behaviour change intervention scenario.	Michie *et al.*, 2020b
Behaviour change intervention scenario	A combination of attributes that are critical to understanding the intervention effects, such as the behaviour change techniques employed (intervention content), the way in which these techniques are delivered (intervention delivery), and the population and setting targeted (intervention context).	Michie *et al.*, 2020b
Entity	Anything that exists, that can be a continuant or an occurrent as defined in the Basic Formal Ontology.	Arp *et al.*, 2015
Ontology	A standardised framework providing a set of terms that can be used for the consistent annotation (or “tagging”) of data and information across disciplinary and research community boundaries.	Arp *et al.*, 2015

Smoking cessation was selected as the first ‘proof of concept’ domain within the HBCP because it is considered to have a higher number of high-quality trials and more homogeneous outcome measures compared to other behavioural domains ( Michie *et al.*, 2017). Over 500 smoking cessation intervention evaluation reports were ***annotated*** using a code set of more than 200 entities from the BCIO, providing a detailed description of each smoking cessation ‘intervention scenario’. The vision for the Knowledge System is that it can automatically scan the scientific literature on smoking cessation and incorporate data from new intervention reports, using information extraction algorithms developed and trained on data from the human-annotated studies ( Michie *et al.*, 2020a). Once the key information is extracted from a given intervention report into the Knowledge System, data are readily available to be exported to different evidence synthesis systems for relevant stakeholders to use. For example, see the HBCP’s Outcome Prediction ( https://pred.hbcptools.org/interface/) and Study Findings and Research Browser ( https://www.humanbehaviourchange.org/browser) proof-of-concept tools. Prediction means that when users ask questions about scenarios of interest to them, the Knowledge System considers subsets of previously annotated entities based on their similarity with the scenario proposed (e.g., behaviour change techniques, population, etc) and predicts an outcome value accordingly (e.g., 9% quit rate at 6 months post-intervention). For further information on the HBCP’s information extraction and prediction approach, see Bonin *et al.*, 2020a, Bonin *et al.*, 2020b, West *et al.*, 2023a and Hastings *et al.*, 2023.

In summary, the HBCP’s ambition is to provide a fast, inexpensive and evidence-based system to extract information from study reports and provide inferences on the potential success of behaviour change interventions, facilitating accumulation and implementation of knowledge. One of the key research questions for the HBCP is to assess whether its ontology-based evidence synthesis methodology can be extended across different behavioural domains ( Michie *et al.*, 2020a). To this end, a second corpus focused on physical activity intervention evaluation reports was developed as part of the project. Physical activity was selected as the next behaviour to provide a contrast with attributes already found in smoking cessation intervention evaluation reports. For example, physical activity studies tend to measure the adoption or increase of a behaviour, rather than abstinence or reduction. In addition, physical activity is thought to be a more complex domain than smoking cessation, with a wider range of outcome measures and behavioural targets.

The aims of this paper are to: (i) describe the development of a corpus of evidence relating to physical activity and (ii) evaluate the extent to which the methods developed for annotating smoking cessation intervention reports were generalisable over this second HBCP corpus. The research questions were:

a) How well did the BCIO-based code set developed for annotating smoking cessation intervention reports work for annotating physical activity intervention reports, and
b) What changes were needed to the code set?

The paper can also serve as a guide to help others when creating a body of evidence to automate evidence synthesis in behavioural science.

## Methods

Building the HBCP physical activity corpus took place in four stages:

### Stage 1: Reviewing the suitability of smoking cessation codes already used in the Human Behaviour-Change Project

Smoking cessation intervention reports were annotated according to a pre-defined, BCIO-informed code set, developed through annotations of reports and discussions with the study team, including an international expert in smoking cessation (RW). The suitability of the smoking cessation annotation code set was initially examined to assess the degree of modifications needed for application to the second behaviour of physical activity. Upon preliminary review the research team identified that smoking cessation codes relating to behavioural outcome would require substantial adaptation. These included:

*Outcome (behaviour)* captured the smoking behaviour defined to be targeted in a given intervention, comprising of four sub-levels: (i) *Behaviour*, specifying the overall behaviour addressed (e.g., *Tobacco use*), (ii) *Behaviour change type*, specifying the type and direction of intended behaviour change for the smoking cessation intervention (e.g., *abstinence, reduction* and *quit attempt*), (iii) *Follow-up*, specifying whether smoking behaviour post-intervention was assessed as a *one-off assessment* or *repeated assessment* and the length of this follow-up, and (iv) *Behaviour assessment*, specifying the type of smoking behaviour assessment, including *subjective assessment* in self-report and informant verification and *objective assessment* in biochemical verification and observation of smoking behaviours. Last, *Outcome (behaviour) value* captured the reported values of the defined smoking outcome (behaviour) for each intervention group (e.g., % abstinent), while *Effect* captured the effect size reported in the intervention evaluation report comparing outcome behaviour values between intervention groups, including the *Effect size type* (e.g., Odds Ratio), *p value* and *95% confidence intervals*.

The adaptation of the above codes for the physical activity corpus involved the following two steps.

*1.1 Identifying behavioural outcomes measured in 100 physical activity behaviour change randomised controlled trials included in Cochrane Reviews*

One hundred randomised controlled trial reports of physical activity behaviour change interventions were annotated to identify the variety of behavioural outcomes they contained. All intervention reports were identified from published Cochrane reviews of physical activity ( Baker *et al.*, 2015; Dobbins *et al.*, 2013; Freak-Poli *et al.*, 2013; Richards *et al.*, 2013) and sedentary behaviours ( Downing *et al.*, 2018; Shrestha *et al.*, 2019), and a meta-analysis of behaviour change techniques in physical activity interventions for inactive adults ( Howlett *et al.*, 2019). We included reviews on sedentary behaviour as part of the scoping work because, while sedentary behaviour is a distinct behaviour within the domain of physical activity, they form part of the same energy expenditure continuum and in many studies – particularly those using accelerometers – both are reported. Information on ‘what’, ‘when’ and ‘how’ physical activity outcomes were assessed in these intervention reports and extracted onto a standardised Excel sheet.

*1.2 Seeking feedback from international experts in physical activity*

Twenty-three international experts in physical activity research were invited to give feedback on the physical activity behavioural outcome codes resulting from the previous step. Experts included 14 behavioural scientists and public health stakeholders that had previously been invited to advise on the HBCP, and nine additional stakeholders identified by the project team. An online questionnaire was emailed to experts in 2018 using Qualtrics XM ^TM^ software (free alternatives include Google Forms or LimeSurvey; we chose Qualtrics as we considered it to be the superior option from a technical standpoint). The survey was designed to be completed within 20 minutes and was divided into six categories (full survey available as online supplementary material 1; West *et al.*, 2023b):

1. *Outcome (behaviour)*: Information about the type of behaviour involved (e.g., time spent engaging in moderate-to-vigorous physical activity 6 months after the start of the intervention).
2. *Behaviour change type*: The type of behaviour change targeted by the intervention (e.g., increase, decrease).
3. *Follow-up*: Information about the assessment made after either an intervention was initiated or an intervention was completed (e.g., 3 months after baseline).
4. *Behaviour assessment type*: The method by which data on the outcome behaviour is collected (e.g., self-report).
5. *Outcome (behaviour) value*: Information about the actual value for this behaviour reported in the study (e.g., mean of 2.3 hours per day).
6. *Effect*: Information about the difference between a given intervention condition and a comparator (e.g., mean difference of 20.1 minutes per day, SD of 12.6, 95% confidence interval 10.3-30.4).

Experts were asked whether they thought any codes should be changed or added within each category and, if so, which ones should be changed or added. The responses were collated by two researchers (EN & EHa), with feedback combined where applicable (i.e., merging similar responses by different experts) and discussed internally by the research team. Revisions were made to the physical activity behavioural outcomes specified in the annotation code set.

### Stage 2: Defining the selection criteria and scope of the corpus

Stage 1 provided a comprehensive overview of the different physical activity behavioural outcomes used in the scientific literature and informed adaptations into the HBCP physical activity annotation code set. Stage 2 entailed discussions between the HBCP’s computer science team and physical activity domain experts within the behavioural science team to narrow down the scope of the physical activity corpus. After establishing the general scope, the process of specifying the inclusion and exclusion criteria for the physical activity corpus was iterative, with the selection criteria expanded and refined as new intervention evaluation reports were reviewed.

In addition, by the time the annotation process for the physical activity corpus started, a series of technical advances implemented in EPPI-Reviewer 4 – a web-based software program used by the HBCP for managing and analysing data ( Thomas *et al.*, 2020) – facilitated a more comprehensive annotation process compared to the first corpus (smoking cessation). These technical advances were discussed and informed further changes to the physical activity annotation code set. An open alternative to this software used for annotation is PDFAnno. We used EPPI-Reviewer because the research team had already expertise and training with this software and the developers were willing to change it to support our needs at no additional cost.

### Stage 3: Identifying and screening physical activity behaviour change intervention reports for inclusion

*3.1 Search strategy*

Physical activity behaviour change intervention reports published in English were searched using Microsoft Academic Graph, one of the biggest, most comprehensive bibliographic databases of scientific literature available at the time ( Visser *et al.*, 2021) which since then has been discontinued (see OpenAlex for a suitable alternative). The search was performed on 20.01.2021 and used the following search string at the title or abstract level: MVPA or “moderate-to-vigorous physical activity” or MPA or VPA or “moderate physical activity” or “vigorous physical activity” or “strenuous physical activity” or “hard physical activity”, with reports additionally filtered using the Microsoft Academic’s built-in Randomised Controlled Trial classifier. These terms were identified through a scoping search in which the first author (OC) manually scanned 20 physical activity behaviour change intervention reports. Found reports from Microsoft Academic Graph were then exported to the reference management tool EndNote – to facilitate the processes of removing duplicates and finding full texts – and finally to EPPI-Reviewer where reports were annotated.

It is worth noting that the search process differed from a traditional systematic review. It was not the author’s intention to locate all relevant research, but to generate a somewhat random subsample of physical activity behaviour change randomised controlled trials to serve as a training set for the Knowledge System. In addition, given the broad selection criteria, it would be implausible to screen, select and annotate all the available literature. For this reason, a target corpus size was set in the first place and articles were screened for inclusion until that point. The target corpus size was based on the computer science team’s previous experience working within the smoking cessation field, which resulted in an estimation of the minimum number of intervention evaluation reports required to train the Knowledge System to extract key features. More specifically, annotating ~100 papers would theoretically allow (i) evaluating existing information extraction and prediction models (trained in smoking cessation) with a different behaviour, as well as (ii) fine-tuning the smoking cessation models on a fraction of the physical activity studies, and then testing them on the remaining studies.

*3.2 Screening titles, abstracts and full texts*

A total of five reviewers (OC, AW, EHa, EHo, CM) worked in pairs to independently screen the title & abstract of the records identified and assess whether they met the inclusion criteria. In a second step, full-text papers of retained intervention evaluation reports were examined by the same reviewers independently, with any discrepancies resolved with a consensus discussion. Disagreements that could not be resolved by consensus were discussed with the rest of the team in weekly meetings.

### Stage 4: Annotating intervention attributes using a code set of 200+ entities from the Behaviour Change Intervention Ontology

*4.1 Annotation process*

The final step after achieving the target corpus size was to annotate the studies. Within the HBCP context, annotation refers to the process of coding selected parts of intervention reports or other resources to identify the presence of ontology entities (i.e., standardised ‘labels’ or ‘codes’ to describe relevant intervention features). For example, in the sentence *“The mean age of participants was 21”*, the researcher would annotate the text *“21”* with the code *“mean age”*. This provides a machine-readable dataset which can be used to train information extraction and prediction algorithms, potentially increasing efficiency and reducing research waste in behaviour change research ( Michie *et al.*, 2017).

The annotation process followed the same methodology as with the annotation of smoking cessation intervention reports: (i) developing an annotation manual iteratively and in collaboration with computer scientists, which specifies the type of data to be annotated against each code and the correct format (e.g., the amount of text to be included in the annotation), (ii) recruiting and training qualified annotators (e.g., researchers with experience in the behaviour change field), and (iii) assigning small batches of intervention evaluation reports to several pairs of annotators (OC, AW, EHa, EHo, CM), who annotate the reports independently and meet at the end of each batch to discuss any discrepancies between their coding. Where there were discrepancies, annotators were encouraged to consult the manual to determine the ‘correct’ way of annotating the relevant code. If the manual did not have a clear answer to the problem, this was brought to the wider team for discussion during weekly meetings (debriefing), with the manual and/or code set updated accordingly. Changes to the manual were additive (i.e., we did not change the modus operandi but rather expanded the manual’s instructions with some edge cases identified during the annotations). Once the coding was finalised and agreed upon, the data were included in the dataset. The HBCP physical activity annotation manual is available as an online supplementary material ( File 2; West *et al.*, 2023b). Highlighted text indicates an addition to the manual as a result of the debriefings.

*4.2 Physical activity annotation code set*

The code set used to annotate the physical activity behaviour change intervention reports was constructed using relevant entities from the Behaviour Change Intervention Ontology (BCIO; https://www.bciontology.org/) and its development followed the stages described above, using the smoking cessation annotation code set as a starting point. The HBCP physical activity annotation code set is available as an online supplementary material to this paper ( File 3; West *et al.*, 2023b), including a code-by-code comparison with the HBCP smoking cessation annotation code set to highlight their differences.

## Results

### Stage 1: Reviewing the suitability of smoking cessation codes already used in the Human Behaviour-Change Project

Modifications to the codes used to annotate smoking cessation intervention reports were discussed by the study team, with a particular focus on behavioural outcomes as these were deemed to be the most behaviour-specific codes. For example, modification types common in smoking cessation focus on decreasing behaviour in the form of abstinence or quit attempts, whereas physical activity interventions are more commonly designed to initiate, increase, or maintain activity behaviours. Types of behavioural assessment also differ between the behaviours, with device-based assessment in the form of activity monitors (such as accelerometers and pedometers) common in physical activity but not in smoking cessation interventions. These initial ideas for modifications to the annotation code set were elaborated by reviewing 100 published physical activity behaviour change intervention reports.

*1.1 Identifying behavioural outcomes measured in 100 physical activity behaviour change randomised controlled trials included in Cochrane Reviews*

Physical activity behavioural outcomes described in these reports were total weekly minutes of activity (k=32) or sedentary behaviour (k=17), percentage of time spent in light physical activity or moderate-to-vigorous physical activity (k=12) or sedentary behaviour (k=7), number of physically active sessions attended in a week (k=11), number of steps (k=10), and percentage of the sample meeting the physical activity guidelines (k=6). Follow-up post-intervention was reported in 87 papers, with the majority reporting follow-up of 12 months (k=43) or 24 months (k=13). Behavioural assessment was performed by self-reported measurements (k=52), parent-report questionnaires (k=15), observation (k=3), or device-based measurements (k=48), including accelerometers (k=27), pedometers (k=15) and heart rate monitors (k=6). Note some studies reported more than one behavioural assessment and thus the sum of the above numbers do not match with the total number of studies reviewed (i.e., 100).

Considering both team discussions and the above extracted data, the annotation code set was modified as follows:

- *Behaviour type* under *Outcome (behaviour)* was modified to include *Physical activity*, with sub-levels of common intensities (Light, Moderate, Vigorous and Moderate-to-Vigorous) and Sedentary behaviour.
- *Initiation*, *Increase* and *Maintenance* of activity behaviours were added to *Behaviour change type* under *Outcome (behaviour)*.
- *Behaviour assessment type* was modified to add *Observation* (e.g., System for Observing Fitness Instruction Time (SOFIT); McKenzie *et al.*, 1992) and *Device-based assessments* including accelerometer, pedometer, inclinometer, and environmental activity sensor monitoring to capture physical activity at the area level ( Roggen *et al.*, 2010).
- *Unit of measurement* was added under *Outcome (behaviour) value* to capture the specification of measurement (e.g., minutes per day, steps per week).
- Changes to *Effect* included adding *Mean Difference*, *Median Difference*, and *Cohen’s d*, as these were more commonly reported in physical activity interventions.

This initial code set to annotate physical activity behavioural outcomes in intervention evaluation reports was used in the next stage.

*1.2 Seeking feedback from international experts in physical activity*

Of the 23 experts contacted, 11 completed the survey and were based in the UK (n=6), Australia (n=3), Canada (n=1) and South Africa (n=1). Expert responses and how these were addressed by the research team are reported as an online supplementary material ( File 4; West *et al.*, 2023b). A summary of changes as a result of the expert feedback is provided below:

- *Behaviour type* under *Outcome (behaviour)* was modified to update our definition of sedentary behaviour to that of Tremblay *et al*. (2017) and to include *Walking*, as a commonly reported, specific physical activity behaviour.
- *Adherence* was added to *Behaviour change type* under *Outcome (behaviour)*.
- *Behaviour assessment type* was expanded to include *Ecological Momentary Assessment* ( Liao *et al.*, 2016).
- *Observation* was moved to be a higher-level code for assessment, alongside *Subjective assessment* and *Device-based assessment*.
- *Indirect calorimetry* was added as a sub-level of *Device-based assessment*.
- *Outcome (behaviour) value* was expanded by adding *Statistical Adjustments* to capture outcome values that are weighted to improve classification of the data, such as adjustment by gender.
- *Hedges’ g* was added to *Effect size type*.

### Stage 2: Defining the selection criteria and scope of the corpus

The physical activity code set resulting from stage 1 was discussed with the computer science team and a decision was made to narrow down the annotations for ‘outcome (behaviour)’ and ‘behaviour’ subsections to focus on behaviour change interventions targeting moderate-to-vigorous physical activity and reporting it as a continuous variable. This was because incorporating different physical activity outcomes would have resulted in a higher number of intervention evaluation reports and annotations being required for training the Knowledge System to recognise and extract such outcomes, greatly increasing the required corpus size.

Moderate-to-vigorous physical activity was prioritised as it has been the main focus of physical activity and public health efforts during the past decades and has the strongest links with both physical and psychological outcomes, compared to other forms of physical activity such as light intensity physical activity or sedentary behaviour ( Owen *et al.*, 2020). In addition, similar to the smoking cessation corpus, we decided to focus on randomised controlled trials due to their recognition as ‘gold-standard’ for studying intervention effectiveness ( Michie *et al.*, 2017). A complete overview of the selection criteria for the physical activity corpus is available in Table 2.

**Table 2.** Selection criteria for the intervention reports included in the HBCP physical activity corpus.

Inclusion criteria	Exclusion criteria
Population • Any age groups. • Healthy individuals as well as people with physical/mental health conditions.	n/a
Research design • Randomised controlled trials (including pilot RCTs).	Research design • Quasi-experimental trials, protocols, qualitative research and economic or process evaluations.
Study aim • Behaviour change interventions targeting physical activity.	Study aim • Epidemiology studies, secondary analyses, analysis of physical activity correlates.
Outcome • Total moderate-to-vigorous physical activity (MVPA), reported as units of time. • Assessed through self-report and/or device-based measures.	Outcome • Studies focused on steps, total physical activity, light-intensity physical activity or sedentary behaviour. • Studies focused on moderate physical activity only, or that report moderate and vigorous physical activity separately. • Studies focused on specific periods of the day (e.g., MVPA during PE classes only) or specific types of MVPA (e.g., household MVPA, leisure MVPA, transport MVPA). • Studies where MVPA is reported as change scores (i.e., no pre- and post- test values available, just change values from baseline).
n/a	Other • Conference submissions, PhD thesis, pre-prints and/or abstract-only entries. • Studies published in languages other than English. • Studies with more than 8 arms. * • Study reports with physical activity results only available in figures/graphs (i.e., where no numerical data can be extracted / annotated) or rotated tables. *

*The rationale for these selection criteria reflects limitations of the software used to annotate intervention reports (EPPI-Reviewer).

In relation to the technical advances in EPPI-Reviewer by the time the annotation process for the physical activity corpus started, these included:

- The possibility to annotate outcome measures at different time points (i.e., pre, post and follow-up measurements).
- A new way to capture outcome values, incorporating the outcome values in a table, as well as their standard deviation and the number of participants per group. Where this data was available, the EPPI-Reviewer software automatically calculated the effect size(s) for the difference(s) between groups). Because effect size was now automatically calculated, it was no longer manually annotated.

These technical advances were incorporated into the physical activity annotation manual and code set.

### Stage 3: Identifying and screening physical activity behaviour change intervention reports for inclusion.

**Figure 1.**
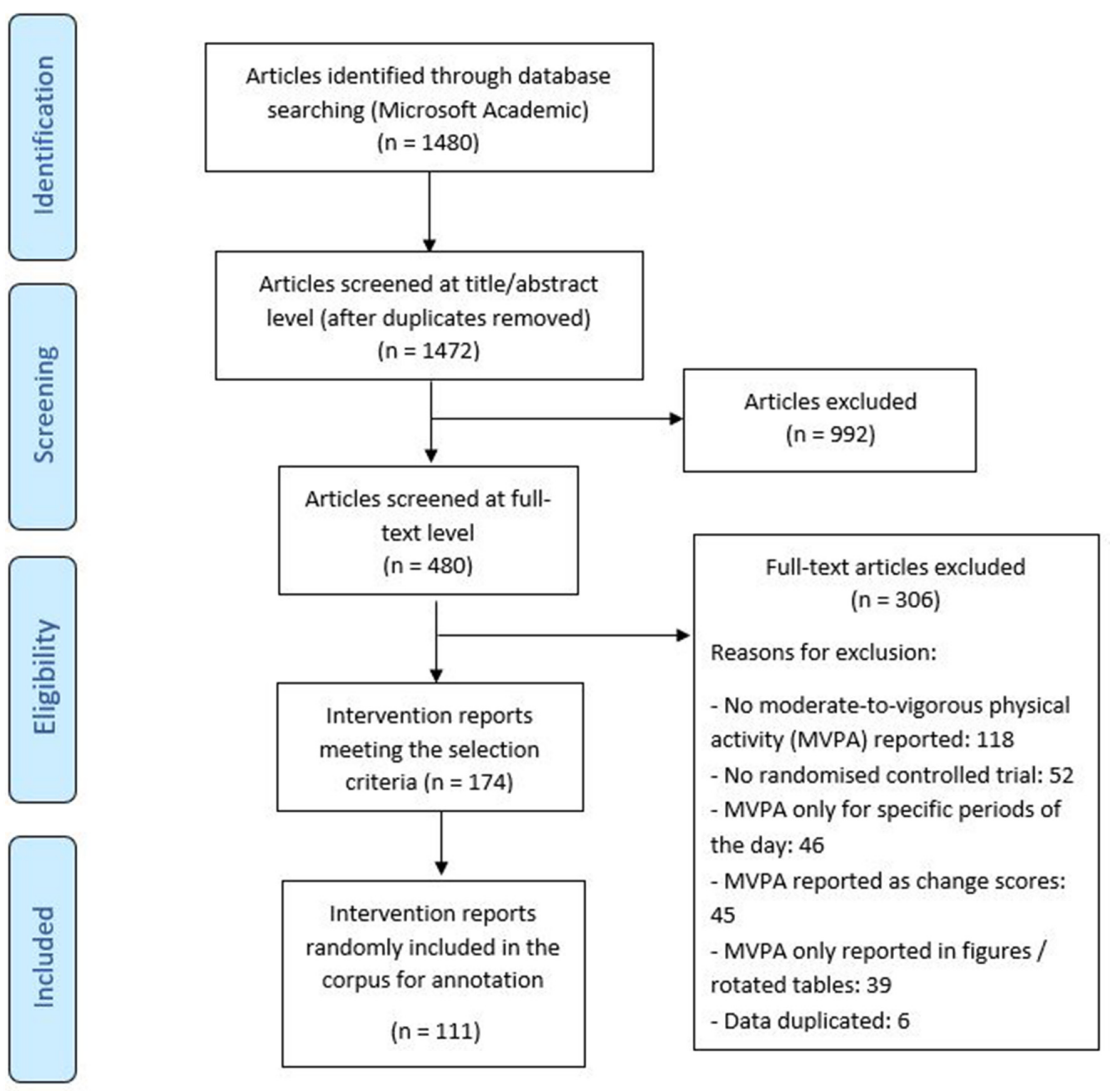
Flow diagram for the intervention reports included in the HBCP physical activity corpus.

A minimum corpus size of ~100 intervention evaluation reports was first established, with batches of articles reviewed for inclusion up to achieving the target ( Figure 1). Because articles were reviewed in batches, the ultimate included sample size was 111 reports.

### Stage 4: Annotating intervention attributes using a code set of 200+ entities from the Behaviour Change Intervention Ontology.

A total of 111 physical activity behaviour change intervention reports published between 2005 and 2020 were annotated (see included reports in online supplementary material 5; West *et al.*, 2023b). Annotations for each of the 111 study reports (JSON file) can be found in the online supplementary materials ( File 6; West *et al.*, 2023b). In addition, the HBCP has developed a Research Browser Tool ( https://www.humanbehaviourchange.org/browser) which allows users to visualise the annotations and use the BCIO to structure queries and locate relevant studies.

The code set was slightly modified as a result of the annotation work and iterative discussions with the research team. This included:

- *Office facility* was added under *Setting* to capture work-based physical activity interventions.
- *Aggregate body mass index (BMI)* was added under *Population* as BMI is particularly relevant for physical activity (e.g., people with high BMI find unique barriers to physical activity) and thus is typically reported in physical activity behaviour change interventions.
- *Funding* and *Competing interests* were expanded to include *Industry with financial interest in intervention success* to capture when physical activity behaviour change intervention studies are supported by makers of devices used in interventions (e.g., Fitbit).

## Discussion

The present paper outlined the steps taken to develop the HBCP corpus of physical activity behaviour change interventions. The main rationale behind developing this corpus was to expand on previous HBCP work and assess the extent to which the methods developed for annotating smoking cessation intervention reports were generalisable over a corpus of evidence relating to a new, and arguably more ‘challenging’ behaviour: physical activity. This is critical to investigate whether the HBCP’s methodology can be applied to improve evidence synthesis across different behavioural domains.

Our process of adapting the annotation code set found that a majority of codes were reusable and can be effectively used to annotate physical activity interventions and potentially other behaviours (e.g., those relating to population, intervention content or mode of delivery). However, findings also underscored certain codes which would require a domain-specific approach, particularly those related to behavioural outcomes such as assessment or modification type. This is consistent with previous literature highlighting the wide variety of outcomes which are relevant to human behaviour ( Larsen *et al.*, 2021; Mallpress, 2022) and suggests that a high degree of granularity is potentially needed within the BCIO to be able to capture these intervention attributes. The currently published BCIO is intended to provide a stable organising structure within which new entities can be added via an open-access portal ( https://www.bciontology.org/contribute), allowing for tracking and “versioning” as the ontology is revised and updated as required.

By creating a second corpus we also hoped to examine whether the information extraction and prediction algorithms developed with smoking cessation studies could be applied to other behaviours. This “transfer learning” – the ability to leverage knowledge gained from one task and apply it to another, often using data from different sources ( Peng *et al.*, 2024) – could enhance the performance of prediction algorithms by integrating evidence from different domains. Additionally, it could reduce the need for extensive human annotations to train information extraction algorithms by reusing pre-existing models, ultimately improving the portability of the HBCP methodology. However, due to variation and ambiguity in the way information is presented in study reports, the information extraction algorithms developed for smoking cessation had limited performance for automated information extraction and for associating information with individual study arms, which posed an insurmountable barrier to full automation ( West *et al.*, 2023a). Therefore, the planned comparisons between the smoking cessation and physical activity domains in terms of accuracy of information extraction and prediction algorithms were not performed. The annotated HBCP corpuses developed for smoking cessation and physical activity could still be used in the future by other research teams pursuing a similar approach to automated evidence synthesis.

### Recommendations for future research

Our approach to creating the HBCP physical activity corpus and adapting the annotation code set to a new behaviour change domain can serve as a guide for those interested in building a corpus of intervention reports for automating evidence synthesis in behavioural science. We include below some recommendations for future research.

First, the groundwork conducted as part of the corpus development process (stage 1) proved crucial to systematically identify and classify relevant outcomes, assess the breath of the field, and help take pragmatic decisions on the corpus’ scope. We recommend undertaking a thorough and systematic outcome identification process for any given behaviour before working towards information extraction automation. Related to this point, a challenge we encountered with our physical activity corpus was the wide variety of different outcomes within the physical activity field. Physical activity is often reported using different variations of physical activity intensities (light, moderate, vigorous or moderate-to-vigorous), but also as number of steps or metabolic equivalents (METs), all using different time frames and frequency metrics such as minutes per day, hours per week, or number of exercise sessions per week ( Sylvia *et al.*, 2014). This makes evidence synthesis in general, and automated artificial intelligence-based evidence synthesis in particular, more difficult. The more heterogeneous a field is, the more data are theoretically required to train an artificial intelligence system working with such behaviour, due to the necessity of having enough examples to ‘teach’ the system how to recognise and extract a given type of outcome entity (e.g., minutes of weekly moderate-to-vigorous physical activity as opposed to number of steps per day). This led us to prioritise a single outcome of interest for our corpus (i.e., moderate-to-vigorous physical activity) and should be considered by future research teams attempting a similar approach for evidence synthesis automation.

An element that may ease future automation attempts in heterogeneous domains, such as physical activity, is to design the Knowledge System in a way that is able to recognise different types of physical activity and perform transformations to the extracted outcomes via pre-specified arithmetic operations. For example, the Knowledge System could be programmed to automatically sum up minutes spent in moderate and vigorous physical activity into a single variable (i.e., moderate-to-vigorous physical activity), improving inter-study operability. Another example would be for the Knowledge System to be able to harmonise the outcomes that are reported in different time frames (e.g., automatically transform hours of moderate-to-vigorous physical activity into minutes or vice versa). The HBCP’s Knowledge System was not developed in a way which allowed these operations.

Regardless of the heterogeneity in outcomes, creating a body of evidence to train information extraction and prediction algorithms will always require finding relevant intervention evaluation reports in the first place. In this regard, we highlight the use of large-scale data sets of scholarly publications (e.g., OpenAlex) as a useful tool to locate intervention reports. Compared to traditional database searching, this approach enables researchers to access a wide range of databases in the same platform, saving time and facilitating automated study identification and incorporation into the Knowledge System, which is key to ensure the system is constantly up to date.

Once the corpus of intervention evaluation reports has been created, it is important to consider that the annotation process takes a substantial amount of time and human resources. Although automated methods hold promise to improve the efficiency of data synthesis over the long term, initial human labour is required to develop and train accurate information extraction algorithms. While annotating intervention reports in pairs is important to ensure high-quality training data, one option if researchers have limited time and resources is to move to single coding once interrater reliability is acceptable and there is a complete, well-developed annotation guide.

Although good annotation tools and processes are important, the production of high-quality training data relies heavily on the data available for annotation. Behaviour change intervention reports, however, tend to use unclear and ambiguous language and this often makes it difficult to accurately interpret and classify data ( Castro *et al.*, 2024; West *et al.*, 2023a). The HBCP found that intervention reports need to be much more structured and consistent in the way they present data. New authoring tools, such as the Paper Authoring Tool (PAT; West, 2020), can be implemented to produce consistent, complete and computer-readable reporting of trials, contributing to improve the extraction and synthesis of data from study reports.

Last, it is also important to recognise that artificial intelligence systems are only as good as the data they are trained on and operate with. The available evidence on behaviour change is not free of bias. For example, successful interventions are more likely to be reported and published ( Nissen *et al.*, 2016). Similarly, most behavioural research is conducted in high-income countries ( Miranda & Zaman, 2010), with predominantly white samples ( Oh *et al.*, 2015). Thus, findings might not be applicable to other contexts and ethnic groups.

### Strengths and limitations

A strength of this research is the systematic, multi-stage process followed to develop the physical activity annotation code set and corpus (including review of intervention reports and international expert consultation) to test the generalisation of annotation methods developed for smoking cessation. Moreover, two researchers independently carried out the screening and annotation of intervention reports, reducing the risk of human error and maximising reliability.

A limitation is the fact that only intervention evaluation reports published in English were considered for inclusion. This means that the annotation methods described here, and the potential information extraction algorithms resulting from such methods, could only be employed with reports in the English language. It is also worth acknowledging that the physical activity corpus size is smaller and has a narrower scope compared to the smoking cessation corpus, meaning findings may not be applicable to all physical activity intervention research. Our intention, however, was not to create a stand-alone corpus but to build upon existing methods applied to smoking cessation intervention reports and use the new domain as a testing ground. Another limitation of this study is the geographical concentration of physical activity experts consulted, primarily from English-speaking countries and the Global North. This focus may limit the generalisability of the findings, as perspectives and contexts from other regions may not be fully represented. As part of the upcoming Advancing Prevention Research In Cancer through Ontology Tools (APRICOT) project – which extends the HBCP work by developing tools for using ontologies in behavioural science ( Michie *et al.*, 2024) – we plan to set up a diverse Community of Practice to ensure broader global engagement in future work.

Last, while this study was not pre-registered due to its largely descriptive nature – lacking hypotheses or analyses susceptible to p-hacking or other questionable research practices – we acknowledge that pre-registration could still have been beneficial (e.g., to enhance discoverability and help prevent unintentional duplication of research efforts). Future hypothesis-driven research conducted using this published corpus, such as training and evaluating information extraction algorithms, should be pre-registered to ensure transparency and minimise publication bias.

## Conclusions

It is possible to generalise the HBCP methods developed for annotating smoking cessation intervention reports to physical activity and potentially other behavioural domains, provided domain-specific groundwork is previously conducted (particularly in relation to behavioural outcomes). This paper provides a blueprint for anyone interested in building a body of research to enhance evidence synthesis in the physical activity field and beyond, including (i) an ontology-informed code set for annotating physical activity behaviour change interventions, (ii) an openly available corpus of 111 annotated physical activity behaviour change interventions which could be used to train and evaluate information extraction algorithms, and (iii) recommendations for future automated evidence synthesis efforts moving forward.

## Ethics

Ethical approval was granted by University College London’s ethics committee (CEHP/2020/579) in February 2020. Participant consent (consultation with physical activity experts) was provided in a dedicated page of the online Qualtrics survey.

## Data Availability

Open Science Framework: Human Behaviour-Change Project. https://doi.org/10.17605/OSF.IO/EFP4X ( West *et al.*, 2023b) Online supplementary materials cited in this article are available below: Supplementary material 1: Questionnaire for physical activity experts ( https://osf.io/9vwye/). Supplementary material 2: HBCP physical activity annotation manual ( https://osf.io/8ekfz). Supplementary material 3: Comparison between HBCP physical activity and smoking cessation annotation code sets ( https://osf.io/n3e9y). Supplementary material 4: Responses to physical activity experts feedback ( https://osf.io/n56kj/). Supplementary material 5: Intervention reports included in HBCP physical activity corpus ( https://osf.io/kdmwe). Supplementary material 6: Annotations for 111 intervention reports included in HBCP physical activity corpus ( https://osf.io/dtn6u). Data are available under the terms of the Creative Commons Attribution 4.0 International license (CC-BY 4.0), which permits unrestricted use, distribution, and reproduction in any medium, provided the original data is properly cited.
