# Supplementary material for: From smoking cessation to physical activity: Can ontology-based methods for automated evidence synthesis generalise across behaviour change domains?
Source: Wellcome Open Res. 2025 Mar 24;9:402. Originally published 2024 Jul 24. [Version 2] doi: 10.12688/wellcomeopenres.21664.2 (PMC11992510; doi:10.12688/wellcomeopenres.21664.2)
Supplement: Peer review history

# Reviewer report: Reviewer response for version 2

**DOI:** 10.21956/wellcomeopenres.26422.r121859

**Published:** 2025-04-11

**Authors:** Neil Howlett

**Affiliations:** 1 Psychology, Sports and geography, University of Hertfordshire, Hertfordshire, UK

This article clearly outlines an approach to build a physical activity corpus with the potential to be helpful to other researchers trying to apply this method, and for future evidence synthesis. The article will be useful to readers and breaks down the process with an appropriate level of detail, which is still accessible enough to those not as familiar with ontologies and the HBCP in general. I note that this is version 2, which has already been revised based on the feedback from two reviewers. I have some minor suggestions, which I hope will add some additional clarity and context.

**Detailed comments:**

 In the second sentence of the plain language summary, the words ‘code’ and ‘use’ should be plural.

 At the end of the second paragraph the current process of data extraction is described as ‘error-prone’. It is clear why it is described as ‘lengthy’ and ‘without a shared conceptual and linguistic framework’, but it is less clear why it is error prone. Does this refer to basic data extraction mistakes from reviewers, inconsistent coding of the same content across review teams (i.e., BCTs), or some other facet? Some context, and ideally a reference to support this assertion, would be useful here. Some commentary, either here or in the discussion, about why the proposed approach will reduce the prevalence of these errors, would be useful too. As already stated in the discussion, intervention reports tend to use unclear and ambiguous language and this often makes it difficult to accurately interpret and classify data. Are there other elements that introduce errors that are specific to manual data extraction, particularly when extraction by two reviewers takes place?

 I note that in version 2 of this manuscript, the authors added justification to stage 1 for including reviews on sedentary behaviour interventions. This is useful context and using an inclusive approach at the stage of screening reviews for behavioural outcomes makes sense. However, I would argue that this is not the same domain as physical activity for three reasons:

- Much of the rationale for exploring the physical activity domain was that this behaviour involves the initiation, increase, or maintenance of behaviour (as opposed to smoking where a reduction or cessation is targeted). Sedentary behaviour interventions target a reduction in the behaviour and, therefore, it could be argued that sedentary behaviour is closer to the smoking domain in terms of the behaviour change type being targeted.
- There is a growing body of research that shows that sedentary behaviour can affect health independently of physical activity levels. For example, there are many people who meet the physical activity guidelines, but that are also highly sedentary (i.e., the rest of their week is spent sitting in front of a laptop at work or television in leisure time).
- Although there are some shared behavioural outcomes between physical activity and sedentary behaviour, they also have ways of measuring these outcomes that are unique (e.g., change in METs per day or week is not used for sedentary behaviour).

I am not suggesting that the method or results should be changed, but there should be an acknowledgement, probably in the discussion, that this approach probably needs to be done again in the future for sedentary behaviour as its own domain distinct from physical activity.

 At the bottom of page 8, it is stated that only RCTs were included because they are a ‘gold standard’. Although this may be true in certain circumstances, RCTs also often represent an unrealistic level of resource and investment in both intervention delivery and evaluation. There is a large body of research that questions whether RCTs are suitable for evaluations of complex public health interventions, and why effects found in RCTs do not consistently replicate in real-world/routine delivery. In addition, most physical activity programmes are not RCTs and are conducted in single-group, non-research designs. Although I understand the need for RCTs in building this corpus, particularly around parameters of effectiveness, the point that these might be delivered in unrealistic conditions should be acknowledged in the discussion. This point would fit well in the last paragraph before the strengths and limitations, where other points of this nature are highlighted.

Is the work clearly and accurately presented and does it cite the current literature?

Yes

If applicable, is the statistical analysis and its interpretation appropriate?

Not applicable

Are all the source data underlying the results available to ensure full reproducibility?

Yes

Is the study design appropriate and is the work technically sound?

Yes

Are the conclusions drawn adequately supported by the results?

Partly

Are sufficient details of methods and analysis provided to allow replication by others?

Yes

Reviewer Expertise:

I have extensive research expertise in assessing the content and effectiveness of physical activity interventions, and in behaviour change methodology more broadly.

I confirm that I have read this submission and believe that I have an appropriate level of expertise to confirm that it is of an acceptable scientific standard.

# Reviewer report: Reviewer response for version 2

**DOI:** 10.21956/wellcomeopenres.26422.r121203

**Published:** 2025-04-03

**Authors:** John Downey

**Affiliations:** 1 University of Plymouth, Plymouth, England, UK

Having looked at the revised manuscript and responses to my initial peer review comments, I am happy that the authors have now made explicit the information I needed to ease the concerns I had. For me, this was more to do with articulating key elements rather than actual rigour. I think the discussion is still quite light and am unable to comment on the AI processes. Nonetheless, I think it is a useful addition to the field, and I would welcome leaning on the teams experience for another project I am involved with exploring how AI was can learning from key documents. I would be happy to see this indexed.

Is the work clearly and accurately presented and does it cite the current literature?

Yes

If applicable, is the statistical analysis and its interpretation appropriate?

Not applicable

Are all the source data underlying the results available to ensure full reproducibility?

No source data required

Is the study design appropriate and is the work technically sound?

Partly

Are the conclusions drawn adequately supported by the results?

Yes

Are sufficient details of methods and analysis provided to allow replication by others?

Partly

Reviewer Expertise:

NA

I confirm that I have read this submission and believe that I have an appropriate level of expertise to confirm that it is of an acceptable scientific standard.

# Reviewer report: Reviewer response for version 1

**DOI:** 10.21956/wellcomeopenres.23937.r102652

**Published:** 2024-10-16

**Authors:** John Downey

**Affiliations:** 1 University of Plymouth, Plymouth, England, UK

Thank you for your manuscript exploring the appropriateness of annotations from the HBCP to date when applied the physical activity literature. The on-going work is novel, has utility, and if it achieves its aim would continue to revolutionise behavioural science. To be accessible and acknowledge the current context, I suggest the below amendments to the current version.

 The title lacks accessibility for the uninitiated decreasing its impact and potential relevance outside a certain audience. I would encourage the authors to alter the wording to increase engagement and decrease the esoteric nature of the work.

 Within the abstract some background on why this is important would be useful, instead of quoting the HBCP. Given health behaviours are such an important area and the large potential of the ontology and AI to short cut and improve our understanding of the literature to date, I think some reference to these contributions is needed.  A minor point also relates to the term ‘evidence synthesis’. I think you could unpack that to provide the setting that I lay out in the initial text.

 Within the introduction I would be conscious of using the realist mantra as I am unsure data from intervention studies provide enough mechanistic and contextual data to answer that question. Some transparency on what the AI could do, has done, and is hindered by would also be useful to set the scene. Likewise in the introduction I think the current issues with the behavioural science literature needs acknowledging and some scene setting on why annotations are needed and how it links to AI being able to answer key questions about interventions.

 The methods are comprehensive, and I like the inclusion of supplementary material and reference to previously peer reviewed elements. It does however look pragmatic and is, as such, lacking detail/transparency on how some procedures were undertaken and key decisions made i.e the survey in phase 1 is not discussed and I am left thinking about construct validity, roll out, completion, and the actual thematic analysis process. In phase 2, if the manual didn’t have an answer, where are these changes recorded?

 In my opinion the discussion does not achieve its aim. A greater immersion in the established literature and consultation with key areas that corroborate, help explain key findings, or detail on how this work fills a gap is needed.

Is the work clearly and accurately presented and does it cite the current literature?

Yes

If applicable, is the statistical analysis and its interpretation appropriate?

Not applicable

Are all the source data underlying the results available to ensure full reproducibility?

No source data required

Is the study design appropriate and is the work technically sound?

Partly

Are the conclusions drawn adequately supported by the results?

Yes

Are sufficient details of methods and analysis provided to allow replication by others?

Partly

Reviewer Expertise:

I am interested in the application of behavioural science by health professionals to support physical activity in those with long term conditions. More recently I am doing applied research exploring digital transformation in healthcare. I would consider myself a behavioural and implementation scientist with an interest in evaluation methodology in complex systems.

I confirm that I have read this submission and believe that I have an appropriate level of expertise to confirm that it is of an acceptable scientific standard, however I have significant reservations, as outlined above.

# Response

**Published:** 2025-03-10

**Authors:** Oscar Castro

**Affiliations:** Future Health Technologies, Singapore-ETH Center, Singapore, Singapore

***Reviewer’s comment**: Thank you for your manuscript exploring the appropriateness of annotations from the HBCP to date when applied the physical activity literature. The on-going work is novel, has utility, and if it achieves its aim would continue to revolutionise behavioural science. To be accessible and acknowledge the current context, I suggest the below amendments to the current version.*

**Response** : We are grateful for the time and energy you expended on our behalf. In the following sections, we will respond to each of your points and suggestions.

***Reviewer’s comment**: The title lacks accessibility for the uninitiated decreasing its impact and potential relevance outside a certain audience. I would encourage the authors to alter the wording to increase engagement and decrease the esoteric nature of the work.*

**Response** : We have rephrased the title to better catch the reader’s attention and highlight the main study aim (i.e., evaluate the transferability of HBCP’s ontology coding methods developed for smoking cessation interventions to the physical activity domain):

- Old title: Creating a body of physical activity evidence to test the generalisation of annotation methods for automated evidence synthesis.
- New title: From smoking cessation to physical activity: Can ontology-based methods for automated evidence synthesis generalise across behaviour change domains?

***Reviewer’s comment**: Within the abstract some background on why this is important would be useful, instead of quoting the HBCP. Given health behaviours are such an important area and the large potential of the ontology and AI to short cut and improve our understanding of the literature to date, I think some reference to these contributions is needed.  A minor point also relates to the term ‘evidence synthesis’. I think you could unpack that to provide the setting that I lay out in the initial text.*

**Response**: We have amended the background section of the abstract to strengthen the study rationale: “Developing behaviour change interventions able to tackle major challenges such as non-communicable diseases or climate change requires effective and efficient use of scientific evidence.”

 However, we are constrained in the abstract due to word count limits, so we have further addressed the reviewer’s comments (i.e., unpack the term “evidence synthesis”) in the introduction: “Evidence synthesis refers to the process of compiling relevant studies on a specific topic to answer…” (paragraph 2).

***Reviewer’s comment**: Within the introduction I would be conscious of using the realist mantra as I am unsure data from intervention studies provide enough mechanistic and contextual data to answer that question. Some transparency on what the AI could do, has done, and is hindered by would also be useful to set the scene. Likewise in the introduction I think the current issues with the behavioural science literature needs acknowledging and some scene setting on why annotations are needed and how it links to AI being able to answer key questions about interventions.*

**Response** : We have amended the introduction to explain the need for an approach such as the HBCP’s and to expand on the role of annotations in the proposed evidence synthesis approach. See new paragraph 2:

 “Evidence synthesis refers to the process of compiling relevant studies on a specific topic to answer a particular research question (Langlois et al., 2018). In recent decades, researchers have placed a stronger emphasis on rigor and accountability of evidence synthesis methods – given birth to the now-standard systematic review methodology – and developed new approaches (e.g., meta-analysis) to statistically combine results from multiple studies (Thomas, 2024). These advancements have undoubtedly benefited behavioural science and other fields, for example, to help ground decisions on scientific evidence (Signore & Campagna, 2023). However, many argue that current evidence synthesis methods have inherent limitations (Moore et al., 2022; Siontis & Ioannidis, 2018) and fail to leverage recent advancements in information and computing sciences which could help address research questions more effectively and efficiently (Sharp et al., 2023; Michie & Johnston, 2017). Behaviour change evidence is currently extracted from study reports through a manual, lengthy, and error-prone process, without a shared conceptual and linguistic framework in the field to facilitate true cumulative knowledge. This leads to research waste (Roberts & Ker, 2015), as findings cannot be easily integrated with other research, and represents a missed opportunity to advance our understanding of behaviour change for the better.”

 In response to reviewer 1, we have also elaborated on the HBCP’s information extraction approach and how it is positioned within the scope of existing work (introduction) and on the limitations of an AI-based evidence synthesis approach (discussion).

***Reviewer’s comment**: The methods are comprehensive, and I like the inclusion of supplementary material and reference to previously peer reviewed elements. It does however look pragmatic and is, as such, lacking detail/transparency on how some procedures were undertaken and key decisions made i.e the survey in phase 1 is not discussed and I am left thinking about construct validity, roll out, completion, and the actual thematic analysis process. In phase 2, if the manual didn’t have an answer, where are these changes recorded?*

**Response** : We have added further details in phase 1 regarding the survey for physical activity experts (see paragraph below). To summarise:

 •    We have removed the word “thematic analysis” as it can be misleading to the reader. Responses from the experts were collated (merging some of them when similar feedback was provided) and then brought to the wider team for discussion.

 •    We have clarified the deployment (roll out) happened in 2018.

 •    There was no construct validity testing as the purpose of the survey was to gather expert feedback on our preliminary list of physical activity behavioural outcome codes (i.e., the survey did not aim to measure a specific concept or theoretical construct).

 “Twenty-three international experts in physical activity research were invited to give feedback on the physical activity behavioural outcome codes resulting from the previous step. Experts included 14 behavioural scientists and public health stakeholders that had previously been invited to advise on the HBCP, and nine additional stakeholders identified by the project team. An online questionnaire was emailed to experts in 2018 using Qualtrics XM TM software (free alternatives include Google Forms or LimeSurvey; we chose Qualtrics as we considered it to be the superior option from a technical standpoint). The survey was designed to be completed within 20 minutes and was divided into six categories (full survey available as online supplementary material 1; West et al., 2023b): (…) Experts were asked whether they thought any codes should be changed or added within each category and, if so, which ones should be changed or added. The responses were collated by two researchers (EN & EHa), with feedback combined where applicable (i.e., merging similar responses by different experts) and discussed internally by the research team. Revisions were made to the physical activity behavioural outcomes specified in the annotation code set.”

 In relation to the physical activity annotation manual, we have clarified in the main text that the changes performed to the document during the annotations: “were additive (i.e., we did not change the modus operandi but rather expanded the manual’s instructions with some edge cases identified during the annotations).”

 We have also highlighted in the annotation manual (File 2 supplementary materials) the portions that were added as a result of this process. For example, during the annotations debriefs there were some uncertainties on how to annotate the number of participants included in studies that use “intention-to-treat”, so further clarifications were added to the manual in this regard (page 32).

 Last, it is worth clarifying that the annotation stage resulted in changes to the manual but (most importantly) also resulted in changes to the physical activity annotation code set (e.g., Office facility was added to capture work-based physical activity interventions). These are reflected in the paper (results section, stage 4). 

***Reviewer’s comment**: In my opinion the discussion does not achieve its aim. A greater immersion in the established literature and consultation with key areas that corroborate, help explain key findings, or detail on how this work fills a gap is needed.*

**Response** : We have amended the first part of the discussion (paragraphs 1-3) to better emphasise the knowledge gap our study aims to fill and to include a wider range of literature. In total, six citations outside the “HBCP ecosystem” have been incorporated into the discussion. However, as indicated to reviewer 1 in relation to the introduction, we are not aware of any other efforts in behavioural science that seek to use ontologies for the automated extraction and synthesis of behaviour change interventions (we have confirmed this by conducting a rapid search through both Elicit and SCITE, using the prompt: “summarise initiatives that leverage ontologies for evidence synthesis in behavioural science”) and thus it is challenging to find analogous work to contrast our approach and findings.

# Reviewer report: Reviewer response for version 1

**DOI:** 10.21956/wellcomeopenres.23937.r100769

**Published:** 2024-10-09

**Authors:** Thomas Gültzow

**Affiliations:** 1 Open University of the Netherlands, Heerlen, The Netherlands

The current manuscript provides a valuable and detailed outline, along with a tutorial, on how to design a Human Behaviour-Change Project (HBCP) corpus. The manuscript is well-written and offers a robust framework for other scholars to follow in conducting similar work. However, there are several areas that could be refined to improve the clarity, transparency, and overall impact of the article.

**Below are my key comments and suggestions for your consideration:**

**Major Comments**

**Citations and Scope**: The citations are heavily focused on the same research team, which is understandable given that the work builds on previous outputs. However, this might create the impression of an isolated 'echo chamber' in which the work was conducted.

**Suggestion**: You may want to consider integrating more citations from external sources and related works outside of the core team to situate your research within the broader scientific landscape. Including more critical perspectives might also enrich the discussion.

**Transparency in Methodology**: While the openness in sharing materials is commendable, some elements of the research process remain somewhat vague, such as the exact search strings used. Also the work does not seem to be preregistered.

**Suggestion**: It may enhance the transparency of the manuscript to provide more detail on each aspect of the process. If proprietary software was used, including a brief rationale for choosing it over open alternatives could help clarify its use, even though the highlighting of open software for others is a great approach. It would also be helpful to provide clarity on whether preregistration was considered (or done), and if not, to explain the rationale behind this decision.

**Inclusion of Sedentary Behaviour**: The inclusion of sedentary behaviour in the study is not clearly justified from the outset. I have always understood that physical activity experts view sedentary behaviour as a separate, albeit related, behaviour to physical activity. Without a clear rationale, readers may be left unclear on its relevance to the study.

**Suggestion**: Offering a clear rationale for including sedentary behaviour early in the manuscript could improve reader understanding.

**Geographical Representation of Experts**: The experts involved seem to be *predominantly* based in English-speaking countries and the Global North.

**Suggestion**: If this was a conscious choice, or if it reflects a limitation, you might consider discussing how this geographical focus could affect the generalisability of your findings. This could also highlight potential avenues for broader global engagement in future work.

**Diversity of the Knowledge Base**: While the manuscript addresses the clarity of the intervention reports as a limitation, it overlooks other critical issues, such as the lack of diversity in the sources. This omission could pose significant challenges in the future, particularly when it comes to the effectiveness of interventions for marginalised populations.

**Suggestion**: Expanding on the limitations related to diversity, as well as other relevant factors beyond clarity, would help provide a more comprehensive discussion of the strengths and limitations of your work. This could also offer a clearer understanding of how these limitations might affect the generalisability of your findings.

**Minor Comments**

**Erroneous Bracket**: In the sentence: “The BCIO offers a set of definitions for describing key entities of behavioural interventions and the casual and semantic relationship between those entities, covering intervention content (Corker et al., 2022; Marques et al., 2023), engagement, population, setting (Norris et al., 2020), target behaviour (Schenk et al., 2024), mechanisms of action (Schenk et al., 2023), and delivery (including mode (Marques et al., 2020), source (Norris et al., 2021), style (Wright et al., 2023) and schedule).” there appears to be a misplaced bracket towards the end.

**Suggestion**: I would remove the bracket at the end of the sentence.

Is the work clearly and accurately presented and does it cite the current literature?

Partly

If applicable, is the statistical analysis and its interpretation appropriate?

Not applicable

Are all the source data underlying the results available to ensure full reproducibility?

Yes

Is the study design appropriate and is the work technically sound?

Yes

Are the conclusions drawn adequately supported by the results?

Yes

Are sufficient details of methods and analysis provided to allow replication by others?

Partly

Reviewer Expertise:

Behavioral and decision-making science focusing on inclusivity, inequalities, and facilitating societal transitions.

I confirm that I have read this submission and believe that I have an appropriate level of expertise to confirm that it is of an acceptable scientific standard, however I have significant reservations, as outlined above.

# Response

**Published:** 2025-03-10

**Authors:** Oscar Castro

**Affiliations:** Future Health Technologies, Singapore-ETH Center, Singapore, Singapore

***Reviewer’s comment**: The current manuscript provides a valuable and detailed outline, along with a tutorial, on how to design a Human Behaviour-Change Project (HBCP) corpus. The manuscript is well-written and offers a robust framework for other scholars to follow in conducting similar work. However, there are several areas that could be refined to improve the clarity, transparency, and overall impact of the article.*

**Response** : Thank you for your effort in reviewing the manuscript and for your constructive feedback. We answer to your queries below.

***Reviewer’s comment**: Below are my key comments and suggestions for your consideration:*

*Major Comments*

*Citations and Scope: The citations are heavily focused on the same research team, which is understandable given that the work builds on previous outputs. However, this might create the impression of an isolated 'echo chamber' in which the work was conducted.*

*Suggestion: You may want to consider integrating more citations from external sources and related works outside of the core team to situate your research within the broader scientific landscape. Including more critical perspectives might also enrich the discussion.*

**Response** : Aside from the Human Behaviour Change Project (HBCP), we are not aware of any other projects in behavioural science that aim to leverage domain-specific ontologies for the automated extraction and synthesis of behaviour change interventions (we have confirmed this by conducting a rapid search through both Elicit and SCITE, using the prompt: “summarise initiatives that leverage ontologies for evidence synthesis in behavioural science”). As a result, most citations refer to prior HBCP work.

 However, we acknowledge there is a wider body of literature on automated information extraction of behavioural science research that does not use an ontology-based approach. We have briefly addressed this in the introduction to better contextualise the HBCP within the broader research landscape:

 “(…). This ontology-based approach distinguishes the HBCP from other information extraction efforts in behavioural and social sciences, which typically rely on different approaches such as distant supervision (e.g., Wei et al., 2022), classifiers (e.g., Zielinski & Mutschke, 2017), or neural networks (e.g., Shen et al., 2022) and are mostly focused on extracting data from abstracts (Legate et al., 2024; Schmidt et al., 2023), likely due to their greater availability and ease of access compared to full texts.”

 In response to reviewer 2 we have also expanded the introduction to provide a stronger rationale for the HBCP approach and this led to the inclusion of several citations beyond the HBCP (e.g., see new paragraph 2). In total, 12 citations outside the “HBCP ecosystem” have been incorporated into the introduction.

***Reviewer’s comment**: Transparency in Methodology: While the openness in sharing materials is commendable, some elements of the research process remain somewhat vague, such as the exact search strings used. Also the work does not seem to be preregistered.*

*Suggestion: It may enhance the transparency of the manuscript to provide more detail on each aspect of the process. If proprietary software was used, including a brief rationale for choosing it over open alternatives could help clarify its use, even though the highlighting of open software for others is a great approach. It would also be helpful to provide clarity on whether preregistration was considered (or done), and if not, to explain the rationale behind this decision.*

**Response** : The keywords stated in the manuscript are all the terms used during the search. We have clarified this in the revised version and also specified the level in which these terms were searched (i.e., title and abstract):

 “(…). The search was performed on 20.01.2021 and used the following search string at the title or abstract level: MVPA or “moderate-to-vigorous physical activity” or MPA or VPA or “moderate physical activity” or “vigorous physical activity” or “strenuous physical activity” or “hard physical activity”, with reports additionally filtered using the Microsoft Academic’s built-in Randomised Controlled Trial classifier.”

 It is worth noting that our search was different from a traditional systematic review search. Microsoft Graph does not work like a standard academic database such as PubMed or Web of Science (e.g., note the use of the RCT in-built classifier (filter) in combination with the search terms) and our goal was to generate an extensive (but not comprehensive) subsample of RCTs on physical activity behaviour change to serve as a training set for the Knowledge System.

 We have added further details in the manuscript to justify the use of the proprietary software, including:

 -    EPPI-Reviewer: “(…). We used EPPI-Reviewer because the research team had already expertise and training with this software and the developers were willing to change it to support our needs at no additional cost.

 -    Qualtrics: “(…). An online questionnaire was emailed to experts in 2018 using Qualtrics XM TM software (free alternatives include Google Forms or LimeSurvey; we chose Qualtrics as we considered it to be the superior option from a technical standpoint).”

 Regarding pre-registration, given the descriptive nature of this work we deemed it as not necessary (i.e., there is no hypothesis or analyses reported which could be susceptible to p-hacking or HARKing). However, we recognise that pre-registration could still have been valuable (e.g., to aid discoverability and avoid duplication of research efforts) and we have included this as a study limitation:

 “(…). Last, while this study was not pre-registered due to its largely descriptive nature – lacking hypotheses or analyses susceptible to p-hacking or other questionable research practices – we acknowledge that pre-registration could still have been beneficial (e.g., to enhance discoverability and help prevent unintentional duplication of research efforts). Future hypothesis-driven research conducted using this published corpus, such as training and evaluating information extraction algorithms, should be pre-registered to ensure transparency and minimise publication bias.”

***Reviewer’s comment**: Inclusion of Sedentary Behaviour: The inclusion of sedentary behaviour in the study is not clearly justified from the outset. I have always understood that physical activity experts view sedentary behaviour as a separate, albeit related, behaviour to physical activity. Without a clear rationale, readers may be left unclear on its relevance to the study.*

*Suggestion: Offering a clear rationale for including sedentary behaviour early in the manuscript could improve reader understanding.*

**Response** : We have provided a stronger rationale for the inclusion of sedentary behaviour, based on the fact that both outcomes are typically measured and reported in physical activity studies:

 “(…). We included reviews on sedentary behaviour as part of the scoping work because, while sedentary behaviour is a distinct behaviour within the domain of physical activity, they form part of the same energy expenditure continuum and in many studies – particularly those using accelerometers – both are reported.”

***Reviewer’s comment**: Geographical Representation of Experts: The experts involved seem to be predominantly based in English-speaking countries and the Global North.*

*Suggestion: If this was a conscious choice, or if it reflects a limitation, you might consider discussing how this geographical focus could affect the generalisability of your findings. This could also highlight potential avenues for broader global engagement in future work.*

**Response** : We have added this as a limitation in the discussion section and elaborated on how we plan to mitigate geographical bias in upcoming work:

 “Another limitation of this study is the geographical concentration of physical activity experts consulted, primarily from English-speaking countries and the Global North. This focus may limit the generalisability of the findings, as perspectives and contexts from other regions may not be fully represented. As part of the upcoming Advancing Prevention Research In Cancer through Ontology Tools (APRICOT) project – which extends the HBCP work by developing tools to facilitate the use of ontologies in behavioural science (Michie et al., 2024) – we plan to set up a diverse Community of Practice to ensure broader global engagement in future work.”

***Reviewer’s comment**: Diversity of the Knowledge Base: While the manuscript addresses the clarity of the intervention reports as a limitation, it overlooks other critical issues, such as the lack of diversity in the sources. This omission could pose significant challenges in the future, particularly when it comes to the effectiveness of interventions for marginalised populations.*

*Suggestion: Expanding on the limitations related to diversity, as well as other relevant factors beyond clarity, would help provide a more comprehensive discussion of the strengths and limitations of your work. This could also offer a clearer understanding of how these limitations might affect the generalisability of your findings.*

**Response** : We agree with this point and that it has not been sufficiently elaborated in the initial version. Despite its potential advantages, an ontology-based evidence synthesis approach will only be as good – or as bad – as the underlying evidence, which is not free of bias. This is a limitation shared with systematic reviews and other “traditional” evidence synthesis methods which potential users should be aware of. We have added the following text and citations to the discussion section:

 “Last, it is also important to recognise that artificial intelligence systems are only as good as the data they are trained on and operate with. The available evidence on behaviour change is not free of bias. For example, successful interventions are more likely to be reported and published (Nissen et al., 2016). Similarly, most behavioural research is conducted in high-income countries (Miranda et al., 2010), with predominantly white samples (Oh et al., 2015). Thus, findings might not be applicable to other contexts and ethnic groups.”

***Reviewer’s comment*** *: Minor Comments*

*Erroneous Bracket: In the sentence: “The BCIO offers a set of definitions for describing key entities of behavioural interventions and the casual and semantic relationship between those entities, covering intervention content (Corker et al., 2022; Marques et al., 2023), engagement, population, setting (Norris et al., 2020), target behaviour (Schenk et al., 2024), mechanisms of action (Schenk et al., 2023), and delivery (including mode (Marques et al., 2020), source (Norris et al., 2021), style (Wright et al., 2023) and schedule).” there appears to be a misplaced bracket towards the end.*

*Suggestion: I would remove the bracket at the end of the sentence.*

**Response** : We have removed the bracket and slightly rephrased the sentence for clarity.
